# *Tinea capitis*: observations and clinical approach in a pediatric population of 99 cases^⋆^

**DOI:** 10.1016/j.abd.2023.03.008

**Published:** 2023-11-18

**Authors:** Carolina Gonçalves Contin, Gustavo de Sá Menezes Carvalho, Guilherme Camargo Julio Valinoto, Silvia Assumpção Soutto Mayor, John Verrinder Veasey

**Affiliations:** Dermatology Clinic, Irmandade da Santa Casa de Misericórdia de São Paulo, Hospital Central, São Paulo, SP, Brazil

Dear Editor,

Scalp ringworm or *Tinea capitis* (TC) is a dermatophytosis that affects both the scalp and the hair shaft.^1^, ^2^ The main described causative agents of TC are from the genera *Microsporum* and *Trichophyton* and the frequency of each pathogen varies depending on the geographic location, environmental and cultural factors of each region and period studied.^3^

The clinical presentation of TC depends on the interaction between the causal agent and the host clinical response, resulting in conditions that vary from mild desquamation with mild hair loss to large inflammatory and pustular plaques.^2^, ^3^ TC can be clinically classified as tonsuring or inflammatory. While the tonsuring form is subdivided into microsporic and trichophytic, the inflammatory form is subdivided into suppurative (or kerion) and favic^2^, ^3^, ^4^ (Fig. 1).

**Figure 1:**
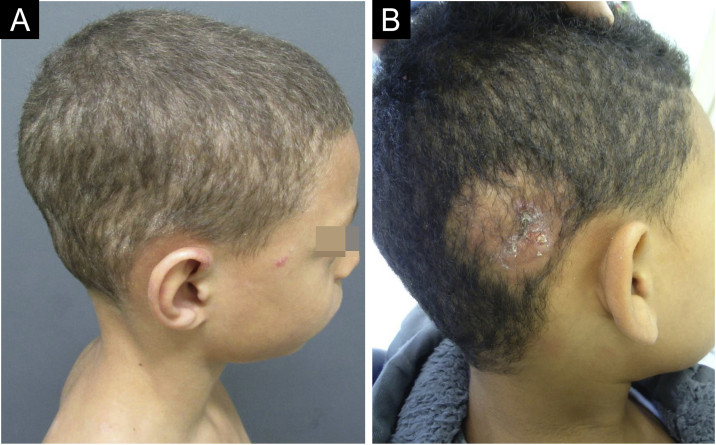
(A) *Tinea capitis*, *Trichophyton tonsurans* clinical type. (B) Tinea capitis, *Kerion celsi* inflammatory clinical type.

TC treatment is based on the use of terbinafine or griseofulvin, with no clinical evidence to support the use of other oral antifungals.^1^, ^4^, ^5^ Griseofulvin was the first effective drug used in the treatment of TC and is still widely used in places with few resources.^1^, ^5^, ^6^ Terbinafine has shown a good safety profile so far and is a good alternative for the treatment of *tinea capitis* in children.^4^, ^5^, ^6^, ^7^

Considering these TC treatment possibilities, a retrospective observational study was carried out with the analysis of data obtained from the medical records of patients with TC treated at a tertiary outpatient clinic in the city of São Paulo (Brazil) between March 2013 and October 2020.

The following inclusion criteria were used: cases with clinical and laboratory TC diagnosis (direct mycological examination and/or positive culture for fungi); those who completed the treatment until clinical and laboratory cure was achieved; those who signed the Free and Informed Consent Form (TCLE, *Termo de Consentimento Livre e Esclarecido*). Patients over 18 years of age, those with incomplete medical record data, those who were lost to follow-up during treatment, and those who did not sign the TCLE were excluded.

During the given period, 148 patients with clinical suspicion of TC were treated. Of these, 99 met the inclusion criteria. Of the 49 excluded cases, two were over 18 years old and 47 patients were lost to clinical follow-up.

The ages of these patients ranged from one to 15 years, with a median of six years. Seventy-five (75.8%) patients were male and 24 (24.2%) were female. The duration of the lesion ranged from one to 192 weeks, with a mean of 15.06 ± 22.72 and a median of eight weeks. Treatment length was up to 40 weeks, with a mean of 14.09 ± 6.87 weeks and a median of 12 weeks (Table 1).

**Table 1:** Absolute and relative frequencies of the clinical form, direct mycological examination, culture and treatment.

Variable		n	%
Clinical type	Trichophytic	56	56.5
	Microsporic	15	15.2
	*Kerion*	28	28.3

Direct mycological examination	Positive	97	98.0
	Negative	2	2.0

Culture	*T. tonsurans*	39	39.4
	*T. rubrum*	2	2.0
	*T. mentagrophytes*	2	2.0
	*M. canis*	34	34.4
	*N. gypseum*	3	3.0
	Negative	19	19.2

Treatment	Griseofulvin	81	81.8
	Terbinafine	5	5.1
	Griseofulvin + Terbinafine	13	13.1

When dividing the total of 99 cases into groups by clinical manifestations, there was a predominance of cases with trichophytic TC, with 56 patients (56.6%), followed by the kerion form in 28 patients (28.3%) and, finally, 15 patients (15.1%) with microsporic TC (Table 2).

**Table 2:** Descriptive values of study variables according to the clinical type group.

	Clinical Form			
Variable	Trichophytic (n = 56)	Microsporic (n = 15)	*Kerion* (n = 28)	p
Age (in years)				0.063^d^
Mean ± SD	6.95 ± 2.80	5.33 ± 1.80	5.71 ± 3.39	
Gender – n (%)				0.028^a^
Female	8 (14.3)	5 (33.3)	11 (39.3)	
Male	48 (85.7)	10 (66.7)	17 (60.7)	
Duration of lesion (in weeks)				0,078^c^
Mean ± SD	18.60 ± 28.71	14.20 ± 13.25	8.96 ± 9.26	
Median	12.00	12.00	8.00	
Direct mycological examination – n (%)				1.000^b^
Positive	55 (98.2)	15 (100.0)	27 (96.4)	
Negative	15 (1.8)	0 (0.0)	1 (3.6)	
Culture – n (%)				0.005^b^
*T. tonsurans*	20 (35.7)	4 (26.7)	15 (53.6)	
*T. rubrum*	1 (1.8)	1 (6.7)	0 (0.0)	
*T. mentagrophytes*	0 (0.0)	0 (0.0)	2 (7.1)	
*M. canis*	25 (44.6)	4 (26.7)	5 (17.9)	
*N. gypseum*	0 (0.0)	0 (0.0)	3 (10.7)	
Negative	10 (17.9)	6 (40.0)	3 (10.7)	
Treatment – n (%)				0.118^b^
Griseofulvin	44 (78.6)	15 (100.0)	22 (78.6)	
Terbinafine	5 (8.9)	0 (0.0)	0 (0.0)	
Griseofulvin + Terbinafine	7 (12.5)	0 (0.0)	6 (21.4)	
Duration of treatment (in weeks)				0.165^c^
Mean ± SD	13.71 ± 7.26	16.27 ± 7.05	13.68 ± 5.92	
Median	12.00	14.00	12.00	

a Descriptive probability level of the Chi-Square test.

b Descriptive probability level of Fisher's exact test.

c Descriptive probability level of the Kruskal-Wallis non-parametric test.

d Descriptive probability level of the Analysis of Variance for one factor.

The analysis of cases according to the isolated agents was carried out in two groups: Group T with the anthropophilic species *T. rubrum*, *T. mentagrophytes* and *T. tonsurans*, and Group M with the non-adapted species (geophilic and zoophilic) *N. gypseum* and *M. canis*. It was observed that most cases (43) were included in the first group, followed by 37 in the second and 19 with negative cultures (Table 3).

**Table 3:** Descriptive values of study variables according to the culture group.

	Culture			
Variable	T Group (n = 43)	M Group (n = 37)	Negative (n = 19)	p
Age (in years)				0.325^d^
Mean ± SD	6.84 ± 2.93	5.86 ± 2.74	6.21 ± 3.19	
Gender – n (%)				0.338^a^
Female	12 (27.9)	6 (16.2)	6 (31.6)	
Male	31 (72.1)	31 (83.8)	13 (68.4)	
Duration of lesion (in weeks)				0,435^c^
Mean ± SD	16.33 ± 31.01	13.65 ± 13.72	15.00 ± 12.00	
Median	8.00	12.00	12.00	
Clinical type – n (%)				0.060^a^
Trichophytic	21 (48.8)	25 (67.6)	10 (52.6)	
Microsporic	5 (11.6)	4 (10.8)	6 (31.6)	
*Kerion*	17 (39.6)	8 (21.6)	3 (15.8)	
Direct mycological examination – n (%)				0.173^b^
Positive	43 (100.0)	35 (94.6)	19 (100.0)	
Negative	0 (0.0)	2 (5.4)	0 (0.0)	
Treatment – n (%)				<0.001^b^
Griseofulvin	28 (65.1)	35 (94.6)	18 (94.7)	
Terbinafine	3 (7.0)	1 (2.7)	1 (5.3)	
Griseofulvin + Terbinafine	12 (27.9)	1 (2.7)	0 (0.0)	
Duration of treatment (in weeks)				0,687^c^
Mean ± SD	11.33 ± 6.26	13.51 ± 6.35	14.68 ± 9.14	
Median	12.00	12.00	12.00	

a Descriptive probability level of the Chi-Square test.

b Descriptive probability level of Fisher's exact test.

c Descriptive probability level of the Kruskal-Wallis non-parametric test.

d Descriptive probability level of the Analysis of Variance for one factor.

When analyzing these two groups regarding the performed treatment, a statistically significant difference (p < 0.001) was observed in the predominance of cases treated with griseofulvin (94.6%) in group M compared to 65.1% in group T, and 27.9% of cases in group T treated with griseofulvin + terbinafine, compared to 2.7% in group M. However, there was no difference in the length of treatment between the two groups.

The proportion of agents isolated in culture, with a predominance of cases related to *T. tonsurans,* reinforces data published by Peixoto et al.,^8^ which raises the discussion about the change in predominance of *T. tonsurans* over *M. canis* in the Southeastern region of Brazil, where the authors’ Dermatology Service is located. The present study also shows the lack of relationship between the agent isolated in culture and patients clinical aspect.^9^ This was observed until 2021 when Meneses et al.^10^ associated trichoscopy patterns in TC with agents isolated in culture. After this analysis, it was possible to determine with greater precision which parasite was likely to be found in each case, instituting a more effective treatment before isolating the agent in the culture.

This recent discovery, together with the perception of change in the prevalence of the etiological agent, explains the disproportion between the three treatment groups: griseofulvin, terbinafine, and griseofulvin + terbinafine. Therefore, the majority of cases included herein belong to the group treated with griseofulvin (81.8%), followed by 13.2% of the group initially treated with griseofulvin who had the antifungal drug changed to terbinafine, and only 5% of cases who received terbinafine from the beginning.

In the clinical practice of the service where the study was carried out, the use of griseofulvin has always been preferred for the treatment of TC. As mentioned, there was an increase in cases of *T. tonsurans* over the years which led to a change of antifungals during patient follow-up, from griseofulvin to terbinafine, after isolation of the fungus in culture. In cases where trichoscopy disclosed specific findings of a certain fungal agent, the appropriate antifungal was administered even before culture isolation, allowing a more appropriate early treatment.

Global studies show that terbinafine is more effective in cases related to the *Trichophyton* genus, while griseofulvin is superior in the treatment of TC caused by fungi of the *Microsporum* genus.^6^, ^7^ In the present study, it was not possible to make comparisons between the medication used and treatment length for each isolated fungal species due to the small number of cases that used terbinafine alone. However, when grouping the agents into group M and group T, there was a statistically significant difference (p < 0.001), with a predominance of cases treated in group M with griseofulvin (94.6%) compared to 65.1% in group T with this antifungal, and 27.9% of cases in group T treated with griseofulvin + terbinafine, compared to 2.7% in group M treated with this association. Despite the above reservations, there was no difference in treatment duration between group M and group T, but this will be better investigated in this service in the coming years.

## Financial support

None declared.

## Authors' contributions

Carolina Gonçalves Contin Proença: Drafting and editing of the manuscript; collection, analysis, and interpretation of data.

Gustavo de Sá Menezes Carvalho: Drafting and editing of the manuscript; collection, analysis, and interpretation of data.

Guilherme Camargo Julio Valinoto: Drafting and editing of the manuscript; collection, analysis, and interpretation of data.

Silvia Assumpção Soutto Mayor: Drafting and editing of the manuscript; collection, analysis, and interpretation of data.

John Verrinder Veasey: Approval of the final version of the manuscript; design and planning of the study; drafting and editing of the manuscript; collection, analysis, and interpretation of data; effective participation in research orientation; critical review of the literature; critical review of the manuscript.

## Conflicts of interest

None declared.

## References

[1] Gupta A.K., Mays R.R., Versteeg S.G., Piraccini B.M., Shear N.H., Piguet V. (2018). Tinea capitis in children: a systematic review of management. J Eur Acad Dermatol Venereol..

[2] Pires C.A.A., Cruz N.F.S., Lobato A.M., Sousa P.O., Carneiro F.R.O., Mendes A.M.D. (2014). Clinical, epidemiological, and therapeutic profile of dermatophytosis. An Bras Dermatol..

[3] Hay R.J. (2017). Tinea capitis: current status. Mycopathologia..

[4] González U., Seaton T., Bergus G., Jacobson J., Martínez-Monzón C. (2007). Systemic antifungal therapy for tinea capitis in children. Cochrane Database Syst Rev..

[5] Fleece D., Gaughan J.P., Aronoff S.C. (2004). Griseofulvin versus terbinafine in the treatment of tinea capitis: a meta-analysis of randomized, clinical trials. Pediatrics..

[6] Gupta A.K., Bamimore M.A., Renaud H.J., Shear N.H., Piguet V. (2020). A network meta-analysis on the efficacy and safety of monotherapies for tinea capitis, and an assessment of evidence quality. Pediatr Dermatol..

[7] Bar J., Samuelov L., Sprecher E., Mashiah J. (2019). Griseofulvin vs terbinafine for paediatric tinea capitis: When and for how long. Mycoses..

[8] Peixoto R.R.G.B., Meneses O.M.S., da Silva F.O., Donati A., Veasey J.V. (2019). Tinea capitis: correlation of clinical aspects, findings on direct mycological examination, and agents isolated from fungal culture. Int J Trichol..

[9] Veasey J.V., Muzy G.S.C. (2018). Tinea capitis: correlation of clinical presentations to agents identified in mycological culture. An Bras Dermatol..

[10] Meneses O.M., Donati A., Silva F.O., Mimiça M.J., Machado C.J., Veasey J. (2023). Trichoscopy patterns of tinea capitis and their correlation with mycological culture results. J Am Acad Dermatol..

